# Severe OCD Exacerbation in a Patient with Autism Spectrum Disorder: A Case Report

**DOI:** 10.26502/acmcr.96550370

**Published:** 2021-05-05

**Authors:** Jasmine Gray, Shahrzad Bazargan-Hejazi, Gul Ebrahim, Daniel Cho

**Affiliations:** 1 Department of Psychiatry, Charles Drew University of Medicine and Science, California, USA; 2 Department of Psychiatry, Charles Drew University of Medicine and Science, David Geffen School of Medicine at University of California at Los Angeles (UCLA), California, USA

**Keywords:** Autism, Obsessive-compulsive, Anxiety, COVD-19, Feeding Problem, Adolescent, Treatment

## Abstract

Autism Spectrum disorder (ASD) is a neurodevelopmental disorder characterized by impairments in social communication and social interaction, repetitive and stereotyped behaviors, and/or sensory aberrations. On the other hand, obsessive-compulsive disorder (OCD) is characterized by the presence of obsessions and/or compulsions. In consideration of these distinct pathologies, research suggests that anxiety disorders and OCD are highly prevalent in individuals with ASD. This case report will discuss an adolescent patient with ASD and OCD who experiences an exacerbation, most notably, in his symptoms of OCD. We outline the hospital course of a 13 year-old male who ultimately requires nasogastric (NG) tube feeding resulting from an acute worsening in symptoms and refusal of oral intake during the COVID-19 pandemic. The patient demonstrated significant improvement in symptoms following the administration of high-dose selective serotonin reuptake inhibitor (SSRI) and low-dose antipsychotic therapy.

## 1. Introduction

It is well-documented that the COVID-19 pandemic has led to exacerbation of pre-existing psychiatric disorders [1]. Specifically, for patients that were already struggling with contamination obsessions, the advent of the pandemic has led to an increase in obsession and compulsion severity. Furthermore, young subjects with obsessive-compulsive disorder (OCD) may develop new symptoms as well as worsening of pre-existing symptoms [2]. While research suggests that anxiety disorders and OCD are highly prevalent in individuals with autism spectrum disorder (ASD) [3], literature also suggests patients with ASD frequently display selective eating patterns [4]. This paper highlights the impact of an infectious disease pandemic on patients already highly vulnerable to decompensation based on the severity of their underlying disorder(s). We will describe the case of a 13-year-old male with high-functioning ASD, OCD, and prior diagnosis of attention-deficit hyperactivity disorder (ADHD). The patient experienced an exacerbation in symptoms during the COVID-19 pandemic and subsequently required NG tube feeding due to complete refusal of oral intake. The patient reported “I’m afraid to take medications”, and was brought in to the emergency department (ED) by his grandmother due to refusal of all oral intake several days prior to presentation. We outline the patient’s hospital course and eventual improvement after receiving a SSRI plus antipsychotic therapy.

## 2. Case Presentation

Mr. X is a 13-year-old white male who was initially diagnosed with high-functioning ASD at 7 years-old, ADHD, disruptive mood dysregulation disorder (DMDD), and then later with OCD, anxiety, and depression at 12 years-old. The patient met all developmental milestones and was enrolled in special education classes in the 7^th^ grade, prior to hospitalization. The patient had exhibited the following symptoms since he was a small child—rigidity, irritability, impulsivity, agitation, aggression, anger, oppositionality, and persistent worrying, with symptoms becoming more pronounced over time. He demonstrated difficulty with cognitive and executive function, such as trouble following two-step instructions (i.e. putting on his socks and shoes) and difficulty completing fine motor skills (i.e. brushing his teeth). Mr. X eventually began to engage in self-injurious behaviors and verbalize suicidal ideation in addition to displaying anhedonia. Mr. X has over 10 documented psychotropic medication trials (several of which were inadequate trials) and one prior psychiatric inpatient hospitalization as the result of a suicide attempt. Prior to hospitalization, Mr. X received regular follow-up care from an outpatient Psychiatrist and a therapist through an ABA (applied behavior analysis) program.

Mr. X presented to the ED due to decreased oral intake (PO) for the past 10 days. His grandmother reported worsened anxiety and hyperfocus related to contaminated surfaces and objects. Mr. X refused *all* PO intake for 4–5 days, while exhibiting OCD symptoms of fear of touching objects and complaining of “itchy skin” that intensified three weeks prior to presentation. Inciting stressors or triggers were unclear. However, the patient’s mother and grandmother believe it was possible that his anxiety worsened following the family’s visit to Urgent Care around Christmas for COVID-19 testing. They claimed this happened after members of the household developed upper respiratory infection symptoms. Of note, all family members returned with negative COVID-19 test results. At the time of presentation to the ED, Mr. X’s condition was so dire that he required several days of stabilization prior to transfer to the psychiatry ward. While in the ED, he was found to have anorexia, dehydration, and metabolic ketoacidosis secondary to starvation (of note, his Body Mass Index (BMI) <5^th^ percentile). All home medications (oxcarbazepine, risperidone, clonidine) were held. As per consultation psychiatry management, sertraline 12.5 mg was initiated. NG tube feeds were started, as he refused intake by mouth as the result of contamination fears and expressed vague weight-gain concerns to staff. After initial stabilization, NG tube feeding was discontinued upon transfer to the child psychiatry ward. However, it would eventually have to be resumed when he continued to refuse oral intake (with the exception of occasionally drinking few sips of water via facet or bath tub).

### 2.1 Clinical evaluation

Upon initial evaluation in the psychiatric ward, physical examination was unremarkable. Mental status exam was notable for a cachectic male who appeared his stated age, and lying in bed, fully alert and oriented. His thought process was linear on direct questioning, although selectively mute in response to several questions. Patient denied suicidal/homicidal ideation, hallucinations, or delusions. Follow-up lab results revealed elevation in Blood Urea Nitrogen (BUN)/Creatinine (Cr) and prompted further work-up to rule-out intrinsic renal disease. Mr. X was found to have prerenal azotemia (BUN as high as 36 mg/dl and serum BUN/Creatinine ratio >20) as the result of inadequate intake, which eventually resolved following adequate hydration. No further medical interventions were required. Psychotropic medications were administered by NG tube feeding including sertraline, resumed after initial initiation on the Medicine ward, at 50mg daily and up-titrated to 100 mg, as well as olanzapine 5mg at night. Mr. X tolerated these medications without adverse effect. During the beginning of treatment, Mr. X was mostly anxious, irritable, and only cooperative with staff after multiple prompts. He displayed rigid and repetitive patterns of behavior, as he insisted that staff perform specific duties for him, such as covering his hands with his blanket in a particular fashion, or commanding them to handle his food/water in a particular manner. Nursing also reported that he occasionally paced back-and-forth in the bathroom in a ritualized fashion. He was isolative and minimally interactive with staff, remaining in his room during the day despite encouragement to get out of bed. He expressed a desire to consume various foods, although he was unable to do this when given the opportunity. Instead, he opted for NG tube feeding and rarely requested to drink nutritional supplement.

Following roughly two weeks of administration of sertraline 50 mg with gradual titration to 200 mg, Mr. X was notably less irritable and more social with staff. He began to participate in group sessions with peers and was amenable to oral intake of supplemental drinks and medications. Such changes were noted to coincide with improvement in his physiologic condition. Although he began this transition to oral feeds in a gradual manner, (only accepting certain meals by mouth daily), the unintended removal of his NG tube was the impetus for consuming all his intake orally. Ultimately, the patient was transitioned to solid foods before discharge to a residential facility from the hospital. The final medication regimen was maximum dose of sertraline at 200mg, olanzapine 5mg, with the addition of Ativan 0.5mg three times a day as needed (PRN) for anxiety.

## 3. Discussion and Conclusions

This report discusses a severe case of ASD and OCD in an adolescent patient who required NG tube feeding after experiencing a deterioration in symptoms following a recent visit to an Urgent Care during the COVID-19 pandemic. Feeding problems in patients with ASD [5] and patients with OCD [6] have been documented. However, no definitive research exists to detail an effective pharmacologic strategy for the occurrence of *both* in a patient, yet alone, an *adolescent* patient. [7] describes “...in the current literature there is an impressive neglect of comorbidities in clinical trials and treatment approaches for these conditions are still not evidence-based.” Interestingly, although the use of antidepressants to treat comorbid ASDs and anxiety has become prevalent, the *data* supporting their use is sparse [8]. Specifically, as it pertains to Sertraline, the FDA has approved its use to treat a variety of mood and anxiety disorders in adults and children including OCD. However, placebo-controlled studies regarding the efficacy among patients with ASDs have yet to be published [8].

In this case, we identified an effective pharmacologic regimen for a patient who experienced an exacerbation in symptoms that resulted in physiologic impairment requiring gradual rehabilitation through NG tube feeding. The patient demonstrated an impressive gain in function following the use of high-dose sertraline and low-dose olanzapine, with later addition of low-dose Ativan PRN for anxiety after approximately six weeks of psychiatric inpatient hospitalization. The described outcome suggests that this medication regimen may be an effective treatment for adolescent patients with underling ASD who suffer an exacerbation in OCD/anxiety symptoms and experience a co-occurring feeding problem. Nonetheless, further research is needed to demonstrate reliability and consistent efficacy of this regimen and to help clinicians navigate the complexities of these co-occurring diagnoses in adolescents.
